# Autoimmune Dysphagia Related to Rheumatologic Disorders: A Focused Review on Diagnosis and Treatment

**DOI:** 10.7759/cureus.41883

**Published:** 2023-07-14

**Authors:** Mohammed Rifat Shaik, Nishat Anjum Shaik, Jamal Mikdashi

**Affiliations:** 1 Department of Internal Medicine, University of Maryland Medical Center Midtown Campus, Baltimore, USA; 2 Division of Rheumatology and Clinical Immunology, University of Maryland School of Medicine, Baltimore, USA

**Keywords:** rheumatologic disorders, immunotherapy, intravenous immunoglobulin, motility disorders, autoimmune dysphagia

## Abstract

Autoimmune dysphagia is defined as dysphagia caused by autoimmune processes affecting various components of the swallowing process such as muscle, neuromuscular junction, nerves, roots, brainstem, or cortex. These autoimmune causes can be classified into gastroenterological, dermatological, rheumatologic, and neurologic. Rheumatological disorders, such as scleroderma, Sjogren's syndrome, systemic lupus erythematosus, rheumatoid arthritis, sarcoidosis, Behcet's disease, anti-neutrophil cytoplasmic antibody (ANCA)-associated vasculitis, or granulomatosis with polyangiitis, have been associated with dysphagia. Autoimmune dysphagia in the context of rheumatological disorders is particularly significant because it can occur as a sole manifestation or as part of a symptom complex associated with the underlying disorder and often responds to immunosuppressive therapies. However, diagnosing autoimmune dysphagia can be challenging as it requires the exclusion of structural and primary motility disorders through procedures such as endoscopy and manometry.

Early diagnosis is important to improve the quality of life and prevent significant mortality and morbidity. Management focuses on treating the underlying disease activity, and a multidisciplinary approach involving various medical specialties may be necessary to achieve success. This article aims to review the autoimmune rheumatological conditions that can lead to dysphagia and discuss the associated pathophysiological mechanisms. We also outline the clinical clues and laboratory testing methods that facilitate early diagnosis, with the goal of improving patient outcomes through timely intervention and appropriate management.

## Introduction and background

Dysphagia, or altered swallowing function with a delay in liquid or solid bolus transit, is mostly related to a disturbance in a complex and integrated sensorimotor system involving the oropharynx and the esophagus [1]. The causes of dysphagia are intricate and range from anatomic and mechanical causes to motility disorders [2]. A significant proportion of the causes of dysphagia are unknown, but dysphagia is prevalent in neurological disorders like stroke, neurodegenerative disorders like Parkinson's disease, and autoimmune diseases [3].

The term "autoimmune dysphagia" refers to dysphagia triggered by autoimmune cell-mediated or humoral immunological processes affecting the musculature involved with swallowing or the nerves, roots, brainstem, or cortex that innervate these muscles [4]. Dysphagia is often motility-related and requires special attention and a multi-disciplinary approach to facilitate a timely diagnosis and treatment to decrease mortality and morbidity from life-threatening complications such as malnutrition, weight loss, aspiration pneumonia, and other restrictions that can have a devastating effect on the quality of life [5].

Autoimmune dysphagia can manifest as either an acute presentation or a progressive symptom that evolves throughout the course of an autoimmune disorder. It can occur solely or in combination with other symptoms, such as skeletal muscle weakness throughout the body. Moreover, dysphagia can be the only initial symptom that leads to suspicion and the subsequent diagnosis of autoimmune illnesses [4]. It remains a diagnosis of exclusion until structural and other motility abnormalities have been ruled out with the help of endoscopy and/or manometric tests. Determining the autoimmune association in these instances is critical, as immunotherapies have the potential to be effective in alleviating dysphagia [4].

Autoimmune dysphagia related to rheumatological disorders is mostly recognized in the context of inflammatory myopathies, particularly inclusion body myositis, polymyositis, and dermatomyositis [6]. Other disorders presenting with autoimmune dysphagia include but are not limited to scleroderma, Sjogren’s syndrome, systemic lupus erythematosus, rheumatoid arthritis, Behcet's disease, anti-neutrophil cytoplasmic antibody (ANCA)-associated vasculitis, and granulomatosis with polyangiitis. An inflammatory reaction involving the swallowing muscles is characteristic [6].

This review summarizes the autoimmune dysphagia associated with rheumatological disorders with an overview of clinical features and immunotherapeutic treatment approaches.

## Review

Idiopathic inflammatory myositis

Idiopathic inflammatory myositis (IIM) is a class of heterogeneous autoimmune disorders characterized by inflammation of the muscles, resulting in motor weakness and a myriad of extra-muscular symptoms such as skin rashes. It can occur as a paraneoplastic manifestation of an underlying malignancy [7]. It is categorized according to the clinicoseropathological features into four primary subgroups, i.e., inclusion body myositis (IBM), dermatomyositis (DM), immune-mediated necrotizing myopathy (IMNM), and anti-synthetase syndrome (ASS) [8,9].

The European League Against Rheumatism (EULAR) and the American College of Rheumatology (ACR) proposed a new probability-score model in 2017 to differentiate IIM from mimicking conditions and distinguish major subgroups of IIM [10,11]. This model takes into account 16 factors (including age at onset, muscle weakness, skin involvement, laboratory markers such as anti-Jo1 antibodies and muscle enzymes, and muscle biopsy features), assigns a score to each factor, and calculates the probability of having an IIM based on the sum of the scores [10,11]. Patients who fulfill the minimal probability of having IIM (>55%) are further categorized based on the age of onset of the first symptom into juvenile IIM and adult IIM. Adult patients with IIM are subclassified into polymyositis (PM), IBM, amyopathic dermatomyositis (ADM), or DM based on the clinical manifestations and muscle biopsy patterns [9-11].

Dysphagia can occur in all subgroups of IIM, although it is most common in IBM. According to a meta-analysis, the estimated pooled prevalence is 36% and as high as 82% when only low-bias risk studies are included [6]. The European Myositis Registry reported a prevalence of 39% [12]. Evidence shows that an associated malignancy and NXP2 (antinuclear matrix protein 2) antibodies are strongly associated with dysphagia [6]. Given the propensity for silent penetration and aspiration, the presence of these risk factors should warrant flexible endoscopic evaluation of swallowing (FEES) or videofluoroscopy (VFSS), even in individuals who do not report symptomatic dysphagia [13,14].

Dysphagia is predominantly oropharyngeal, involving the striated muscles of the hypopharynx and upper esophageal sphincter (UES). Mechanisms include chronic inflammation, edema, and muscular atrophy [15]. Inflammation may be diagnosed with an esophageal biopsy or through MRI features such as oropharyngeal edema. Four patterns of swallowing impairments have been noted, including impaired pharyngeal contractility, cricopharyngeal failure, diminished laryngeal elevation, and esophageal hypomotility [6,16]. Diminished pharyngeal contractility can lead to inadequate bolus clearance and an increased risk of aspiration [17]. Cricopharyngeal failure can cause UES dysfunction, which can be demonstrated by VFSS as a cricopharyngeal bar, muscular propulsions, or posterior indentations between C3 and C7 [6]. Since the larynx needs to be elevated for the UES to open, any defect in laryngeal elevation may negatively affect UES function [18]. In addition, esophageal motility may be decreased or absent, with the dysfunction occasionally extending to the lower esophageal sphincter (LES) [6].

The four primary subgroups of IIM are discussed below.

Inclusion Body Myositis (IBM)

IBM is the leading cause of debilitating dysphagia related to inflammatory myopathies and is most common in those over the age of 50 [4]. The prevalence of dysphagia is estimated to be 50% per the Euro myositis registry, 56% per meta-analysis, and 40% per retrospective study [6,12,19]. Up to 80% of participants reported symptoms when a targeted questionnaire seeking symptoms of dysphagia was performed in a cohort with IBM [20]. About 10% may present with dysphagia as an isolated clinical feature [4]. For instance, Shibata et al. describe a case report in which a patient with IBM had dysphagia for five years prior to the onset of limb muscle weakness [21]. Dysphagia usually presents at an advanced disease stage, resulting in unsatisfactory treatment outcomes [22].

The presence of anti-CN1A antibodies correlates with severe dysphagia [23]. However, there is a poor correlation between dysphagia and abnormalities observed on the VFSS [22]. In a study by Murata et al., patients without clinical dysphagia exhibited a mild degree of pharyngeal propulsion, while all patients with dysphagia displayed a severe degree of pharyngeal propulsion. Interestingly, all patients who did not have clinical dysphagia were found to have cricopharyngeal achalasia [24].

Mechanisms likely involve UES spasms resulting from cricopharyngeal muscle hyperplasia/hypertrophy or fibrosis, suprahyoid and pharyngeal muscle weakness, and diminished descending bolus forces. Other mechanisms, such as decreased epiglottic deflection, impaired laryngeal elevation, reduced tongue control, and poor base tongue retraction, are also implicated [22].

Immune-Mediated Necrotizing Myositis (IMNM)

IMNM, also known as necrotizing autoimmune myositis (NAM), is the most prevalent inflammatory myopathy across all age groups [25]. It is associated with connective tissue disorders (such as scleroderma), myositis-specific autoantibodies (i.e., anti-SRP and anti-HMGCR), malignancy, viral infections (HIV or hepatitis C), or immune checkpoint inhibitors. A characteristic feature is the absence of primary inflammation on muscle biopsy [26]. Muscle weakness is often more severe, and the disease course is shorter with acute and subacute presentations. A retrospective case study noted ophthalmoparesis, ptosis, and facial muscle weakness as clinical presentations [19]. CK is usually elevated more than five times the upper limit of normal [19].

More than 30% of individuals with NAM in the European Myositis Registry and 28% in a retrospective analysis were documented to have dysphagia [12,27]. In the above-mentioned retrospective study involving five patients, VFSS showed more pharyngeal phase involvement than oral. Cricopharyngeal bar/prominence was demonstrable in one patient [19].

Dermatomyositis (DM)

DM is characterized by the subacute onset of skin changes (heliotrope rash on the upper eyelids, a flat red rash on the anterior chest in a V sign, or on the back and shoulders called the shawl sign, and erythema of the knuckles with a raised, violaceous, scaly eruption described as Gottron rash) and proximal muscle weakness. Mucosal changes such as mucosal edema, erythema and telangiectasia, aphthous stomatitis/ulcer-like lesions, and xerostomia may also be seen [12,28]. It is associated with an underlying malignancy in 15% of the cases [29].

The estimated prevalence of dysphagia is 31% per a meta-analysis and 43% per the European Myositis Registry [6,12]. In a study involving 95 patients with DM, dysphagia was noted in 13 patients (14%). All 13 patients who underwent VFSS demonstrated pharyngeal dysphagia without oral or esophageal dysphagia and had classic symptoms, including pharyngeal pooling and/or nasal regurgitation. Patients with a lower modified muscle testing (MMT) score for their sternomastoid and dermatomastoid muscles and those who were older, male, had an internal malignancy, or had anti-transcription intermediary factor 1 antibodies (anti-TIF-1 Ab) were more likely to experience dysphagia. Interstitial lung disease (ILD) was shown to have a negative association with dysphagia. Patients with dysphagia had higher muscle, cutaneous, and global disease activity [30].

Dysphagia may result from inadequate muscle contraction and reduced hyolaryngeal excursion rather than from a weak UES, as is widely assumed [30]. Associated macroglossia can worsen dysphagia [31].

Anti-synthetase Syndrome Overlap Myositis

ASS is characterized by myositis, ILD, skin rash, arthropathy, and the Raynaud phenomenon. Antibodies against aminoacyl transfer RNA synthetase (anti-ARS), typically anti-Jo-1 antibodies, are typical [32].

Dysphagia was noted in 29% of the patients per the European Myositis Registry and 17% in a single-center study [33]. The severity was mild to moderate in comparison to other IIMs [33].

Systemic sclerosis

Systemic sclerosis (SSc) is a connective tissue disorder of unknown origin characterized by vasculitis and fibrous dysfunction involving multiple organ systems, including the esophagus. Dysphagia primarily results from esophageal dysfunction, with a minor contribution from the oral and pharyngeal phases [34].

Esophageal involvement is dominated by GERD and/or esophageal dysmotility [35]. Esophageal dysmotility affects roughly 20-95% of patients [36]. Some studies demonstrate no difference in esophageal involvement between the diffuse and restricted types of SSc. In contrast, others suggest a higher incidence and severity of esophageal dysmotility with diffuse scleroderma and substantial skin involvement [34,37]. The antibodies anti-Scl 70 and anti-RNPC-3, but not anti-centromere, have been associated with dysmotility [38]. About 35% of people with dysphagia also have gastroesophageal reflux, although only around 4% of the population with SSc experiences dysphagia [39]. Muscle fibrosis and atrophy develop over time due to initial vascular dysfunction, followed by neurogenic dysfunction [40]. Distal two-thirds of the esophagus is affected, resulting in LES dysfunction. The characteristic manometric findings include a decrease in LES pressure and the presence of aperistalsis or ineffective peristalsis in the distal esophagus, referred to as classic scleroderma esophagus [35,36]. An increase in esophageal transit time and gastroesophageal reflux can also be noted. Over time, even mild cases of peptic esophagitis may worsen into more serious cases marked by erosive esophagitis, bleeding, and ulcers. Long-term, untreated GERD can lead to complications, including Barrett's esophagus and esophageal adenocarcinoma [34].

In addition, the oral and/or pharyngeal phases of swallowing may also be affected. Microstomia and limited mandibular movement can cause oral dysphagia by impairing mastication and the ability to form a food bolus. Oral phase challenges may be exacerbated by concomitant Sjogren's syndrome (SS), if present. Pharyngeal dysphagia may result from myositis, stenosis, or weakness of the cricopharyngeal muscles [34].

Sjogren’s syndrome (SS)

It is a lymphocyte-mediated, infiltrative autoimmune disorder characterized by the destruction of exocrine glands, predominantly the lacrimal and salivary glands, resulting in sicca symptoms such as xerophthalmia and xerostomia [41].

About 32-71% of SS patients experience some degree of dysphagia, with as many as 40% reporting severe dysphagia [41]. In a study by Mandl et al. involving 20 patients with primary SS and 30 age-matched controls, SS patients displayed much greater rates of dysphagia than controls (65% vs. 3%). However, pharyngeal and esophageal dysmotility rates were not significantly higher than those of healthy controls [42].

Dysphagia may be oropharyngeal or esophageal. Pharyngeal transit times and tongue contraction against the posterior pharyngeal wall were both shown to be delayed, as was the cricopharyngeal opening. In 35-40% of patients, esophageal dysmotility is seen. There may be a decrease in pressure at the UES, as well as a variety of nonspecific motility problems, including aperistalsis, tertiary contractions, non-peristaltic contractions, and decreased contractility. The peristaltic velocity may be slowed down. The pressure in the LES may be variable, and the sphincter may be shortened [43].

Multiple mechanisms can be implicated, including xerostomia, neuropathy, cricoarytenoid joint arthritis, esophageal dysmotility, and gastroesophageal reflux [44]. Xerostomia, by interfering with the triggering of peristalsis, could result in delayed swallowing during the oral and pharyngoesophageal phases, decreased acid clearance in the esophagus, and increased symptoms of esophageal dysmotility [45]. Dysphagia may result from complications associated with xerostomia, such as opportunistic infections (Candida albicans), dental caries, and loss of teeth [41]. IgG autoantibody-mediated disruption of muscarinic receptor function and parasympathetic dysfunction, suggested by a lower expiration/inspiration (E/I) ratio, have also been implicated. In addition to regulating esophageal motility, parasympathetic function regulates esophageal and salivary exocrine secretion. Thus, parasympathetic dysfunction may result in dysmotility and reduced salivary and esophageal exocrine secretion, culminating in dysphagia [42].

Mixed connective tissue disorder

Mixed connective tissue disorder (MCTD) is characterized by a high titer of anti-U1-RNP antibodies and any two of the following: edema of the hands, synovitis, myositis, Raynaud's phenomenon, and sclerodactyly [46]. There may be involvement of multiple organ systems as the disease progresses. Gastrointestinal involvement is frequent, with the esophagus being most frequently affected [47].

Dysphagia is caused by esophageal dysmotility. Hypo or aperistalsis of the lower two-thirds of the esophageal body and a hypotensive LES is characteristic [48]. In contrast to the study by Gutierrez et al., which found aperistalsis in more than half of patients, Marshall et al. demonstrated that peristalsis in the proximal esophagus was preserved [49,50].

Marshall et al., in a study involving 61 patients with MCTD, noted symptomatic dysphagia in 38% of the patients. Pressure at the LES and peristaltic pressure amplitude in the lower esophagus was significantly lower than in healthy controls. Out of 35 patients that underwent manometric studies, 17% demonstrated aperistalsis, and 43% had low-amplitude distal peristaltic contractions [49]. Gutierrez et al. by utilizing esophageal manometry in a group of 17 patients found that only one had normal motility; two had segmentally weak esophageal peristalsis (amplitude < 25% of normal); five had aperistalsis of the lower two-thirds of the esophagus, and nine had either aperistalsis or weak peristalsis involving the entire esophagus. Both studies also consistently reported hypotension of the UES [50]. Ten patients who had manometry examinations before and after corticosteroid medication showed a statistically significant improvement in LES pressures following therapy [49].

Systemic lupus erythematosus (SLE)

SLE is a multisystem disorder characterized predominantly by constitutional symptoms of low-grade fever, arthralgias, or arthritis and cutaneous manifestations. It can involve hematologic, musculoskeletal, neuropsychiatric, mucocutaneous, renal, and gastrointestinal systems [51].

About 1 to 13% of the patients demonstrate dysphagia [52]. The primary cause is esophageal dysmotility, affecting 21 to 72% of the patients [53]. This results from inflammation reaction in the musculature, muscular atrophy, or ischemic vasculitic injury to the Auerbach plexus [52].

Upper esophageal involvement occurs in 14% of the cases [53]. Lower esophageal and LES involvement, although less prevalent, hypo- or aperistalsis in the lower esophagus with hypotony of the LES pressure may be seen, especially with concomitant scleroderma or mixed connective tissue disease. This increases the likelihood of gastroesophageal reflux, which worsens dysphagia [52]. The esophagus may become inflamed with ulceration, mucosal bridging, or perforation [52]. A quarter of individuals are affected by oral lesions, mostly on the palate, buccal mucosa, gingiva, and lips which may worsen dysphagia [52,54].

Raynaud's phenomenon appears to be associated with the development of esophageal dysmotility [52]. While Montecucco et al. demonstrated a good correlation between the presence of the Raynaud phenomenon and/or anti-A1 antibodies with esophageal dysmotility, Lapadula et al. showed no such correlation [55,56]. Borrow et al. in a case report proposed an association of anti-PM-Scl antibodies with dysphagia [57].

Rheumatoid arthritis

Rheumatoid arthritis (RA) is a chronic inflammatory joint disease that can cause cartilage and bone damage as well as disability [58].

Oral, pharyngeal, and esophageal motility problems are common in RA patients; 13.1 to 33.3% report dysphagia [59,60]. Oropharyngeal dysphagia can be ascribed to dry mouth, difficulty and pain with mastication and masticatory fatigue, cricoarytenoid joint dysfunction, and pharyngeal segment immobility and dysfunction related to inflammation and destruction of the cervical spine and mandible [59-61]. Patients present with difficulties swallowing solids, taking smaller bites, and chewing excessively for safe swallowing, discomfort in the throat or chest when swallowing, and the sensation of food sticking in the throat [62].

Esophageal dysfunction may be caused by the underlying disease, the medication, or both. Mechanisms include esophageal motor dysfunction, secondary amyloidosis, vasculitis, and/or anti-rheumatic medication-related side effects such as pill-induced esophagitis [63]. Esophageal motility problems affect 30% of individuals [64]. Manometry findings include low-amplitude peristaltic waves in the lower two-thirds of the esophagus and decreased pressure at the LES. These may worsen gastroesophageal reflux disease. Poor peristalsis in the proximal esophagus and delayed esophageal clearance are also described [65,66].

Thyroid impairment was associated with a threefold increase in the prevalence of dysphagia in patients with RA. Hypothyroidism, particularly myxedema, is associated with oropharyngeal symptoms. There have also been reports of lower esophageal dysfunction, such as achalasia, in patients with autoimmunity and hypothyroidism [62,67].

Behcet’s disease

It is a multisystem autoimmune inflammatory disease characterized by oral and genital ulcers and ocular symptoms. Oral and, much less, gastrointestinal system involvement characterizes the illness [68].

The most likely cause of dysphagia includes oral, pharyngeal, or esophageal ulcers. Oropharyngeal dysphagia may also develop in the presence of myositis-related pharyngeal stenosis and pharyngeal webs [69]. These are both very uncommon and documented via case reports. Only 2 to 11% have esophageal involvement, which commonly manifests as esophageal ulcers in the middle or lower esophagus [70]. Esophageal motor dysfunction is prevalent, as evidenced by nonspecific motility abnormalities on the manometry. This is attributed to an abnormality in the ganglion cells of Auerbach and focal degenerative changes in the brainstem disrupting the vagal supply to the esophagus [70]. Also possible are reflux esophagitis, strictures, and perforation [71].

Wegener’s granulomatosis

Involvement of the esophagus can occur as a part of multi-system involvement by vasculitis in Wegener’s granulomatosis. There have been case reports describing the presence of dysphagia, odynophagia, esophageal ulcers, and upper gastrointestinal bleeding [72].

Sarcoidosis

Sarcoidosis is an inflammatory, granulomatous, multisystem disorder that primarily affects the lungs, although it can involve any organ system [73]. Esophageal involvement is rare, and only a limited number of case reports have been documented to date [65]. When the esophagus is affected, the lower esophagus is more commonly involved than the upper esophagus [74]. Dysphagia often occurs as a result of muscular and myenteric plexus involvement [75,76]. Mucosal involvement can lead to esophagitis and potentially cause strictures, further exacerbating dysphagia [77,78]. In some cases, mediastinal lymphadenopathy can directly compress the esophagus and affect swallowing [65,79].

VFSS findings may include a lack of peristalsis in the esophageal body and incomplete opening of the LES after swallowing. Hypertonicity of the UES and narrowing at the pharyngoesophageal junction may also be observed [65,80].

Diagnosis

In order to diagnose autoimmune dysphagia, structural and primary motility disorders need to be ruled out using investigations, including cervical CT scans, endoscopies, barium swallow tests, and manometric studies [81-83]. Each rheumatological condition is associated with distinct abnormal motility patterns, but none are specific to any particular disorder [84]. Diagnosis is often based on a patient's medical history and any concurrent skin and muscle findings. Laboratory studies, such as an autoimmune panel and muscle enzymes, as well as procedures like EMG and muscle biopsy, may be necessary to make a definitive diagnosis [85].

Different healthcare institutions have their protocols for working up dysphagia, but a basic framework is provided in Table 1. The first step in this process is distinguishing between oropharyngeal and esophageal phase abnormalities, typically through a thorough medical history. Symptoms such as difficulty initiating a swallow, coughing, choking, or nasal regurgitation indicate oropharyngeal phase pathology. The onset of dysphagia and whether symptoms are progressive or intermittent can help distinguish structural from motility issues. A thorough physical exam that includes a skin assessment for any demonstrable lesions (such as malar or discoid rash - lupus, heliotrope rash, knuckle rash, V or Shawl sign - DM or Raynaud phenomenon - RA), evaluation of muscle strength, and identifying the muscle groups involved can provide insights into the autoimmune etiology [28,86,87]. Thus, when an autoimmune etiology is suspected, a targeted autoimmune panel including ANA (antinuclear antibodies), anti-dsDNA (anti-double-stranded DNA), anti-Smith, anti-SSA (anti-Sjogren's syndrome A), anti-SSB (anti-Sjogren's syndrome B), RF (rheumatoid factor), anti-CCA (anti-cyclic citrullinated peptide), anti-Scl 70 (antibodies targeting Scl 70: topoisomerase 1), and anti-RNPC-3 antibodies (antibodies against RNA-binding region containing 3) can be obtained. If concomitant proximal muscle weakness is present, a CK and myositis panel, including anti-cN1A (anti cytosolic 5ʹ-nucleotidase 1A), anti-SRP (antibodies against signal recognition particle), anti-HMGCR (antibodies against 3-hydroxy-3-methylglutaryl-coenzyme A reductase), anti-NXP2 (antinuclear matrix protein 2 antibody), anti-Jo1 (antihistidyl t-RNA synthetase antibody), and antiM2 (anti-mitochondrial M2 antibody) can be considered [4]. Definitive diagnosis involves electromyography (EMG) and muscle biopsy [86].

**Table 1:** Work-up for autoimmune dysphagia CT: computed tomography, MRI: magnetic resonance imaging, EGD: esophagogastroduodenoscopy References: [1,45-49]

History and physical examination
Onset, duration and progression
Skin assessment for any lesions
Evaluation of muscle strength for any weakness
Targeted autoimmune panel testing based on the clinical suspicion
Oropharyngeal dysphagia
Cervical CT
MRI head +/- neck
Fiberoptic endoscopic swallowing examination (FEES)
Modified barium swallow study (MBS)
Flexible laryngoscopy
Esophageal dysphagia
Endoscopy (EGD)
Barium swallow study (esophagogram)
High-resolution manometry
EndoFLIP (endoluminal functional lumen imaging probe)
Esophageal transit (scintigraphy)
Cine-sophagraphy

Patients with clinical suspicion of oropharyngeal dysphagia may benefit from a clinical swallow examination (CSE) to evaluate the ability to swallow foods and liquids of varying consistencies. Cervical CT can help exclude obstructive causes, while central neurogenic causes, such as stroke and brainstem lesions, can be ruled out after consultation with a neurologist and an MRI brain [88,89]. If there are any warning signs of malignancy, such as rapid weight loss, dysgeusia, or bleeding, a laryngoscopic evaluation may help screen for tumors and anatomical abnormalities in the oropharynx [82]. If silent aspiration or motility issues are a concern, a fiberoptic endoscopic swallowing examination (FEES) is necessary to assess the throat and pharynx, as well as the swallowing mechanism and secretion management [82]. Another option is VFSS, also called a modified barium swallow study (MBSS), which allows dynamic and real-time visualization of the oropharyngeal and pharyngoesophageal stages of swallowing. The food is coated in barium during this test and viewed using an X-ray or fluoroscopy machine [82,83]. MRI neck is an alternative option that is equally reliable for detecting impaired food passage [90].

Endoscopy and/or barium swallow are valuable tools in the evaluation of esophageal dysphagia. Upper endoscopy (esophagoscopy) enables direct observation of mucosal lesions and tumors and allows for biopsies and therapeutic intervention if required [91]. A barium swallow study (esophagogram) is particularly useful for identifying potential structural causes of dysphagia and is the preferred initial study in those with a history of stricture or a potential proximal esophageal lesion [92]. In cases where symptoms suggest esophageal dysmotility or the results of initial tests are inconclusive, esophageal manometry may be performed. High-resolution manometry (HRM) is considered the gold standard for assessing esophageal motility [81]. However, recent guidelines recommend using a functional luminal imaging probe (FLIP) as a complementary tool to HRM, particularly in cases of manometric esophagogastric junction outflow obstruction (EGJOO) or other inconclusive patterns [93]. Alternative options for evaluating esophageal motility include esophageal transit scintigraphy and cine-esophagography [94].

Treatment

Treatment options can be broadly categorized into behavioral, pharmacological, and invasive therapy. Effective management requires a multidisciplinary approach with input from medical practitioners, occupational therapists, or speech pathologists.

Behavioral Strategies

Behavioral strategies, which include dietary modifications and swallowing therapy, are effective early in the disease course and may even be explored when no effective treatment exists [95]. Compensatory interventions, such as a modified diet, modified feeding techniques (such as taking smaller bites and alternating solids and liquids), and exercises (such as tongue base retraction and an effortful swallow) can improve swallowing function [4]. Excellent dietary recommendations for esophageal dysmotility include taking small bites, thoroughly chewing food, avoiding dry or fibrous meals, ensuring adequate fluid intake with solid foods, and refraining from lying down for an extended period after eating. Specific techniques, such as the Mendelssohn Maneuver and linguistic strengthening program can help patients maintain a stable weight and continue eating without aspiration, particularly in the early stages of dysphagia when some muscle function is still present [19]. Speech pathologists may provide guidance for preventing choking and reducing apprehension of impending aspiration [96].

Pharmacological Therapy

Medical therapy is the foundation of treatment and includes immunotherapy and adjunctive therapy.

Immunotherapy is effective, especially for myositis-associated dysphagia. Numerous case reports and case series support the use of high-dose intravenous corticosteroids as the initial treatment for PM/DM [6]. In a retrospective study involving patients with DM-associated dysphagia, a significant correlation was found between high initial dosages of prednisolone and improvement in dysphagia [97]. Typically, an initial intravenous high-dose pulse of up to 1000 mg methylprednisolone per day for 3-5 days is administered in severe cases, followed by a maintenance dose of prednisolone at 0.5-1.0 mg/kg per day for several weeks. The dose is then gradually tapered, aiming for a maintenance dose of 5 mg per day after six months [98]. Other immunosuppressive treatments like methotrexate, tacrolimus, mycophenolate, cyclophosphamide, rituximab, hydroxychloroquine, and cyclosporine can be added as steroid-sparing agents or in the absence of adequate response to steroids after three months [6]. Immunotherapy is also effective in IMNM and anti-SS-OM, with a more rapid response observed [4]. On the other hand, dysphagia in IBM is refractory to conventional immunosuppressive treatments [19]. For instance, in a retrospective study, only two of 16 IBM patients that received steroid medication reported an improvement in their dysphagia and the effect was only transient [19].

Adjunctive therapy can improve swallowing in certain cases by addressing the underlying cause of dysphagia. For example, in SS, alleviating xerostomia (dry mouth) is key, which can be achieved through artificial saliva or secretagogues to boost salivary flow [43]. Acid suppression therapy is beneficial when peptic lesions contribute to dysphagia such as in scleroderma or lupus [38]. Treatment of underlying malignancy alleviates dysphagia in DM [30]. Overall, the treatment approach should be tailored to the specific underlying condition causing dysphagia.

Role of Immunoglobulin (IVIg)

The emergence of medication resistance or intolerable adverse effects with steroids or immunosuppressants led to studies investigating the role of high-dose intravenous immunoglobulin (IVIg) for IIM. While the results varied, most of the studies showed a significant improvement in dysphagia (Table 2). There is no consensus globally on the role of immunoglobulin for dysphagia, emphasizing the need for high-quality longer duration clinical trials. Some recommend IVIg, along with other immunosuppressants, as first-line therapy for patients with myositis, particularly DM, especially in the presence of ILD and/or dysphagia. However, others reserve it as a second-line therapy or as a third-line add-on therapy for those not well controlled with a combination of steroids and methotrexate or azathioprine and for patients who are immunodeficient or for whom immunosuppressants are contraindicated [96,99,100].

**Table 2:** Studies demonstrating the benefit of IVIg for the treatment of autoimmune dysphagia in various rheumatological disorders VAS: visual analogue scale; EAT: eating assessment tool; FEES: fiberoptic endoscopic evaluation of swallowing; IVIg: intravenous immunoglobulin; ScIg: subcutaneous immunoglobulin; CK: creatine kinase; LDH: lactate dehydrogenase

Study	Type of Study	Disease treated	Number of Patients treated	Duration of Therapy	Results	Additional information
Barsotti et al.	Multicenter retrospective series	IIM	123	Mean duration of 6 months	Improvement in dysphagia (59 vs 23% p<0.001) and VAS scores (42.4 ± 37.2 vs 12.4 ± 21.8 p<0.001)	78% of patients were deemed to have a "responder" status after receiving IVIg. Responders had an older age at the disease onset and a shorter overall duration of the disease. Higher CK and LDH levels prior to treatment were associated with a more favourable response [101].
Giannini et al.	Retrospective study	IIM	12	3 monthly doses	Significant improvement in solid stasis, liquid stasis, EAT-10 and FEES scores	Complete restoration of defective propulsion and a gradual reduction in solid and liquid stasis corresponded with a decrease in the EAT-10 score after three monthly doses of IVIG. All of these parameters remained stable throughout the course of the 52-week follow-up [102].
Marie et al.	Retrospective study	PM/DM	73	Median of 7 months	82.2% had resolution of esophageal problems. Swallowing abnormalities significantly improved within 2 weeks after the first dose with full resolution of esophageal impairment within 5-15 days after the second or third dose.	Criteria for resolution include the complete elimination of clinical symptoms. Criteria for deterioration include worsening of esophageal clinical symptoms [99].
Aggarwal et al.	Clinical trial	Dermatomyositis	94	Up to 40 weeks	Compared to the placebo group, patients in the IVIG group had greater improvement on a composite measure termed the Total Improvement Score at week 16.	Multicenter, double-blind, randomized, placebo-controlled Phase III trial. Every 4 weeks, patients received IVIg or placebo. Those who deteriorated at week 8 switched treatment groups, and those in the IVIg group were dropped from the study. All other patients (those who got placebo or IVIg who didn't deteriorate) entered the Extension Period (weeks 16 to 40) [40].
Cherin et al.	Case series	IBM	6	4.5 to 27 months	Improvement in muscle strength and resolution of dysphagia. Two patients had a 12-month improvement before relapse.	ScIg therapy enables once- or twice-weekly dosing of lower dosages to sustain therapeutic serum IgG levels with enhanced therapeutic effect and less systemic adverse effects [103].
Murata et al.	Case series	IBM	3	IVIg therapy followed by balloon dilation after 3 months	Subjective reports of dysphagia diminished, and the VF analysis showed an increase in the volume of barium paste traversing through the UES.	The participants were able to resume regular meals after balloon therapy. The quantity of barium paste flowing through the upper esophageal sphincter (UES) increased, food residue in the piriform recess decreased, and aspiration with an inflow of barium paste into the larynx decreased [104].
Dobloug et al.	Retrospective study	IBM	12 patients with esophageal dysmotility	Mean of 10 infusions (range: 3-25)	Three IVIg-treated patients described subjective improvement in swallowing function during follow-up compared to none in the control group.	Three patients with severe dysphagia who were treated with IVIg also had surgery or Botox injections. Despite IVIg therapy, repeated dynamic studies in two individuals demonstrated a deterioration of dysmotility [105].
Chaigne et al.	Retrospective study	IBM	10 patients with dysphagia received IVIg		Non-statistically significant improvement in dysphagia was noted [106].	

Barsotti et al. used IVIg to treat 123 IIM patients with active steroid-resistant myositis and/or for contra-indication to immunosuppressive treatment. A total of 71-82% of the patients were noted to demonstrate a subjective improvement in dysphagia and a significant decrease in Visual Analogue Scale (VAS) scores [101]. In a study conducted by Giannini et al., 12 patients with IIM-related dysphagia who had not responded to high-dose glucocorticoids with methotrexate or azathioprine showed a progressive improvement of FEES abnormalities and the reduction in EAT-10 scores at 52 weeks of follow up [102]. In the systematic review and meta-analysis by Goswami et al., intravenous (IVIg)/subcutaneous (SCIg) immunoglobulin (Ig) therapy was found to significantly reduce cutaneous disease activity and dysphagia with a steroid and immunosuppressant sparing effects in about 40% of patients with refractory idiopathic inflammatory myopathy (IIM) and juvenile dermatomyositis (JDM) [107].

Marie et al. published a multicenter series involving the use of IVIG in 73 patients with PM/DM with steroid-resistant esophageal involvement. Swallowing improved significantly in 89% of cases within two weeks, allowing them to return to normal oral feeding, have their feeding tubes ablated, and be rapidly discharged from the hospital. Therefore, they advocated the use of IVIG in severe, steroid-resistant esophageal dysfunction secondary to PM/DM and proposed combined IVIG and high-dose steroid therapy as the first-line treatment [99]. Aggarwal et al. performed a randomized, placebo-controlled trial involving patients with active DM. Patients were given IVIg or a placebo every four weeks for a maximum of 40 weeks. A composite measure called the Total Improvement Score was superior in the IVIG group compared to the placebo group at week 16 [40].

In contrast to other IIMs, dysphagia in IBM is more resistant to steroid treatment. While Cherin et al. suggested some benefits with IVIg in stabilizing severe dysphagia, the Cochrane review by Jones et al. demonstrated no substantial benefit [103,108,109]. In a double-blind, placebo-controlled crossover study with 19 IBM patients with dysphagia, the patients who received monthly infusions of 2 g/kg IVIg/placebo for three months demonstrated a modest, statistically significant improvement in swallowing duration as evaluated by ultrasonography [110]. Murata et al. observed a transient improvement lasting two months with IVIG and balloon dilatation of UES in a group of three IBM patients who were previously unable to consume half-solid meals [104]. Likewise, Dobloug et al. discovered short-term improvements in severe dysphagia in a retrospective study involving 12 patients with esophageal dysmotility [105]. Cherin et al. demonstrated a sustained improvement lasting as long as 12 months using subcutaneous immunoglobulin (SCIg) in six patients with IBM, despite recurrent relapses [103]. Chaigne et al. showed a non-statistically significant improvement in dysphagia and a steroid-sparing effect following one year of treatment in SSc-associated myopathy (SScAM) [106].

IVIg works by inhibiting the production of autoantibodies, fixing complement, neutralizing the autoantibodies or autoantigens, and inhibiting or blocking cytokines. It suppresses T-cell-derived IL-2, IL-10, TNF-α, and IFN-γ and impedes dendritic cell maturation. It inhibits the phagocytosis of antibody-coated cells by blocking Fc-receptors on autoantibodies [28]. It stimulates the expression of the inhibitory Fc and Fc- y- RIIB receptors attenuating autoantibody-induced inflammation. It inactivates the C3 convertase precursor decreasing complement amplification [96,111].

Interventional Therapies

Interventional methods such as cricopharyngeal myotomy, pharyngoesophageal dilatation, and botulinum injection of the UES have been attempted for the management of dysphagia in IBM [112]. These interventions aim to alleviate cricopharyngeal dysfunction and reduce the risk of aspiration, without directly affecting the course of the disease [90]. In a retrospective review of 26 patients with IBM-associated dysphagia, significant improvement in symptoms was observed in 63% of patients who underwent cricopharyngeal myotomy and 30% of those who underwent pharyngoesophageal dilatation [112]. The rationale behind cricopharyngeal myotomy is to lower the pressure exerted by the pharyngeal constrictors, thereby facilitating bolus passage through the esophagus [19]. However, it should be noted that the effects of these interventions are temporary and repetitive treatments may be required to sustain the improvement in symptoms and swallowing function [6].

Data on the use of percutaneous endoscopic gastrostomy (PEG) tubes is conflicting. Some argue that early placement of PEG tubes may be necessary to prevent aspiration pneumonia and malnutrition until immunotherapies start to take effect [4]. However, it is important to note that in the above-mentioned retrospective study, all six patients that underwent PEG tube placement died of aspiration pneumonia. This highlights the need for further research regarding the role of PEG tube placement in this context [112].

## Conclusions

In conclusion, this article provides an overview of autoimmune dysphagia in the context of rheumatological disorders. The pathophysiological mechanisms involved the clinical features, and the existing therapeutic strategies, particularly immunotherapy and IVIg were described. However, it is important to acknowledge that there is still much to be learned about the underlying pathophysiology of autoimmune dysphagia, and further studies are needed to advance our understanding and develop more effective treatment options.
